# Timing and threshold of high sensitive troponin T measurement for the prediction of mortality after cardiac surgery: a retrospective cohort analysis

**DOI:** 10.1186/s40635-023-00545-z

**Published:** 2023-09-01

**Authors:** Andreas Koköfer, Crispiana Cozowicz, Bernhard Wernly, Niklas Rodemund

**Affiliations:** 1 Department of Anaesthesiology, Perioperative Medicine and Intensive Care Medicine, Paracelsus Medical University of Salzburg, Salzburg, Austria; 2 Center for Public Health and Healthcare Research, Paracelsus Medical University of Salzburg, Salzburg, Austria; 3 Department of Internal Medicine, General Hospital Oberndorf, Teaching Hospital of the Paracelsus Medical University of Salzburg, Salzburg, Austria

**Keywords:** High sensitive troponin, Cardiac surgery, Mortality, ROC, Logistic regression modelling

## Abstract

**Background:**

High sensitive cardiac troponin T (hsTnT) is a widely used biomarker of myocardial injury. Along with other high sensitive troponins, HsTnT can predict mortality in both cardiac and non-cardiac surgery. The aim of this study was to determine the association between hsTnT serum elevations in the immediate postoperative period until 120 h after cardiac surgery and the occurrence of in‐hospital mortality compared to the Simplified Acute Physiology Score 3 (SAPS3). Additionally, we identified an ideal hsTnT serum threshold to predict in‐hospital mortality.

**Methods:**

We performed a retrospective single-institutional cohort analysis of 2179 patients undergoing cardiac surgery with cardiopulmonary bypass from 2013 to 2021. Logistic regression analysis was used to investigate an association of hsTnT at various time points and in-hospital mortality. The model was adjusted for relevant covariates including SAPS3, lactate and administered norepinephrine dosage. ROC analysis was performed to estimate the accuracy to predict mortality by serum hsTnT concentrations. This prediction was compared to the SAPS3 score. An ideal cutoff of hsTnT concentration was calculated by means of Youden index.

**Results:**

In total 7576 troponins were measured at the predefined timepoints. 100 (4.59%) patients died during the hospital stay. The fourth hsTnT on d3 (at 96–120 h postoperatively) showed the highest association with in-hospital death (*OR 1.56; 95% CI (1.39–1.76); p* < *0.001).* This finding persisted after multivariable adjustment *(aOR 1.34; 95% CI (1.18–1.53); p* < *0.001)*. In contrast, the third hsTnT on d2 (at 48–72 h postoperatively) showed the best discrimination for in-hospital mortality (*AUC 82.75%; 95% CI (0.77–0.89).* The prediction by the third hsTnT was comparable to the in-hospital mortality prediction by SAPS3 *(AUC 79.36%; 95% CI (0.73–0.85); p* = *0.056).* The optimal cutoff for the third hsTnT was calculated to be 1264 ng/L (*Sensitivity 0.62; Specificity 0.88)*.

**Conclusion:**

Elevated hsTnT after cardiac surgery was associated with an increased risk of in-hospital mortality. HsTnT measured on postoperative day 2 and 3 were most accurate to predict in-hospital mortality. The prediction of in-hospital mortality using hsTNT is comparable to mortality prediction using the SAPS3 score. HsTnT serum levels currently recommended to establish clinically important periprocedural myocardial injury are lower than thresholds identified in this study.

**Supplementary Information:**

The online version contains supplementary material available at 10.1186/s40635-023-00545-z.

## Introduction

More than 1 million adults worldwide die within 30 days of noncardiac surgery each year [1]. Given the increasing demand for cardiac surgery globally a simple and reliable tool to predict postoperative mortality is urgently needed [2, 3]. Currently, preoperative and postoperative risk prediction widely relies on scoring systems like the European System for Cardiac Operative Risk Evaluation (EuroSCORE II/soon III) for risk stratification in cardiac surgery [4] or the Simplified Acute Physiology Score 3 (SAPS3) [5] for ICU mortality. Whereas the EuroSCORE II is designed to predict a priori mortality before any cardiac surgery, the latter is designed and evaluated to predict in-hospital mortality after admission to the ICU. However, all scoring systems have similar downfalls: They are subject to calibration drift as patient populations and surgical techniques evolve and are complex to collect. In addition to scoring systems, biomarkers have recently gained wide popularity in the prediction of risk and mortality in both cardiac and non-cardiac surgery [6]. In this context, troponins are among the most extensively studied biomarkers. As cardiac specific proteins, troponins are released into the circulation by cardiomyocytes upon any (ischemic and non-ischemic) injury to the myocardium. Elevated serum levels of troponin in the blood have been associated with an increased risk of mortality in both non-surgical and surgical populations [7–11]. The use of high-sensitivity (hs) troponin assays has significantly improved the ability to detect even small troponin elevations, which have important implications for the prediction of postoperative mortality. Consequently, this advancement may aid in the identification of high-risk patients [8–10]. Currently, there are two high-sensitivity substrates, troponin I and T (hsTnI/ hsTnT) in clinical use. Elevations in both hsTnI and hsTnT occur after virtually every kind of cardiac surgery, making it difficult to define which threshold indicates increased mortality after cardiac surgery. According to recent studies, currently accepted hsTnI serum thresholds, as established by expert consensus of the Fourth Universal Definition of Myocardial Infarction [12] or the Academic Research Consortium-2 recommendation [13], may be too conservative and unable to accurately identify increased perioperative mortality [10, 14]. It is also unclear if changes within the acceptable threshold in one hs troponin substrate can be extrapolated to the other. Furthermore, there is only limited knowledge on the postoperative kinetics of hsTnT. The timing of troponin measurement in the postoperative course may profoundly affect the ability to predict postoperative mortality. Hence, this study sought to investigate the effectiveness of sequential postoperative hsTnT measurement for the prediction of in-hospital mortality after cardiac surgery using cardiopulmonary bypass (CPB). We hypothesized, that later hsTnT measurements on postoperative day 2 (d2) or 3 (d3) are superior (more accurate) in the prediction of mortality compared to early measurements on the day of surgery (d0) or on the first postoperative day (d1). In contrast to d2 and d3, earlier hsTnT measurements could be more associated and reflective of interoperative adverse occurrences (e.g. reperfusion injury, surgical trauma, defibrillation, etc.) which may per se increase hsTnT levels but not necessarily predict the prognosis of the patient. Additionally, this study aimed to compare the predictive ability of hsTnT as a standalone biomarker for postoperative mortality after cardiac surgery to that of the SAPS3 score. Furthermore, it investigated whether incorporating hsTnT into the SAPS3 score enhances its predictive accuracy for postoperative mortality.

## Methods

### Study design

This retrospective cohort analysis included patients who underwent elective and urgent cardiac surgery utilizing CPB at the General Hospital Salzburg, Paracelsus Medical University from 2013 to 2021. Data was extracted from the institutional Salzburg Intensive Care database (SICdb) which was recently developed [15]. SICdb was implemented using data from the hospital ICU data management system (PDMS) iMDsoft MetaVision ICU (iMDsoft, Needham, MA, USA) and the electronic health record (EHR) ORBIS (DH Healthcare GmbH, Bonn, Germany). The database contains admission, discharge, procedural and ICD10 data. Readmissions of patients recorded in SICdb can't be completely ruled out due to the retrospective analysis methods; however, they are unlikely in the case of cardiac surgeries. Within the dataset, readmissions were not specifically tested for. For each admission, only the first surgery was evaluated. Additionally, all medications (including dosage), procedures such as renal replacement therapy (RRT), intubation etc. are reported in SICdb. All data are fully pseudo-anonymized as defined by the European General Data Protection Regulation [16]. The deidentification strategy additionally complies with the 'Guidance Regarding Methods for Deidentification of Protected Health Information in Accordance with the Health Insurance Portability and Accountability Act (HIPAA) Privacy Rule' [17]. Only patients who are older than 18 years were analyzed. Reporting standards set by the REporting of studies Conducted using Observational Routinely-collected health data (RECORD) initiative were followed (see Additional file 1: Appendix 1) [18]. Patients with missing data on hsTnT levels at the predefined timepoints were excluded. The study was approved by the State Ethic Commission of Salzburg, Austria. (EK Nr: 1115/2021) Given the sole use of de-identified data, written informed consent was not required.

### Endpoints

The primary aim of the study was to identify the ideal postoperative time point of hsTnT measurement for the most accurate prediction of mortality by comparing the AUCs of the individual time points. Serum hsTnT concentrations were measured using the ROCHE Elecsys^®^ Troponin T high sensitive (TnT-hs) assay. The highest hsTnT concentration in each time period was used. Predefined time points for the measurements were: first hsTnT at d0—imminent postoperative period, upon ICU admission, second hsTnT at d1—1st postoperative day at 6am (i.e.: 12–24 h postoperatively), third hsTnT at d2—second postoperative day at 6 am (i.e. 48–72 h postoperatively), fourth hsTnT at d3—third postoperative day at 6am (i.e. 96–120 h postoperatively). Secondary objectives of this study included comparing the predictive ability of hsTnT and the SAPS3 score for in-hospital mortality, as well as determining the optimal hsTnT cutoff for mortality prediction.

### Statistical analyses

We expressed non-normally distributed data points as median ± interquartile range [IQR]. Normally distributed data is given in mean ± standard deviation (SD). Categorical data were stated in numbers (percentage). Univariate distribution differences between groups were calculated using the Chi-square test, Mann–Whitney U test and one-way ANOVA, as appropriate. A univariate model using logistic regression analysis was generated for the primary binary outcome 'in-hospital mortality', (hereafter referred to as Model 1). For each of the 4 hsTnT measurements, a Model 1 was created. Additionally, we corrected Model 1 for potential confounders in a multivariate logistic regression model (hereafter referred to as Model 2). For the multivariable regression model, cofounders with a p-value < 0.10 in the univariate analysis were included, then a backward variable elimination was performed. Elimination criterion was a p-value > 0.10. Based on clinical relevance and literature, variables included in the selection were age, sex, type of procedure (CABP, AVR, and other valve procedures), CPB time, cross-clamp time, average lactate at d0 and d1, and maximum dose of norepinephrine. A Complete Case Analysis (CCA) approach was used for missing hsTnT values. To evaluate the prognostic performance of hsTnT at each time point, we assessed the area under the fitted receiver operating characteristics curve (AUC) using a maximum likelihood logistic regression model. We additionally assessed the corresponding integrated discrimination improvement (IDI) using the SAPS3 score as a reference. The IDI reflects of prognostic performance of hsTnT serum concentrations compared to the reference model and the gain in prognostic performance when adding the hsTnT serum concentrations to the reference model [14]. An optimal cut-off of hsTnT was calculated in the best performing AUC by means of the Youden-Index. The discrimination of the cutoff value was evaluated using Chi-square test and multiple linear regression, respectively. We reported adjusted odds ratios (aOR) with respective 95% confidence intervals (95% CI) for all models. All statistical tests were two-sided, a p-value of < 0.05 was considered statistically significant. The R Project for Statistical Computing (RCore Team, 2022) was used for all statistical analyses. Results were visualized using R Studio (RStudio Team, 2022, Boston, MA, USA).

## Results

**Fig. 1:**
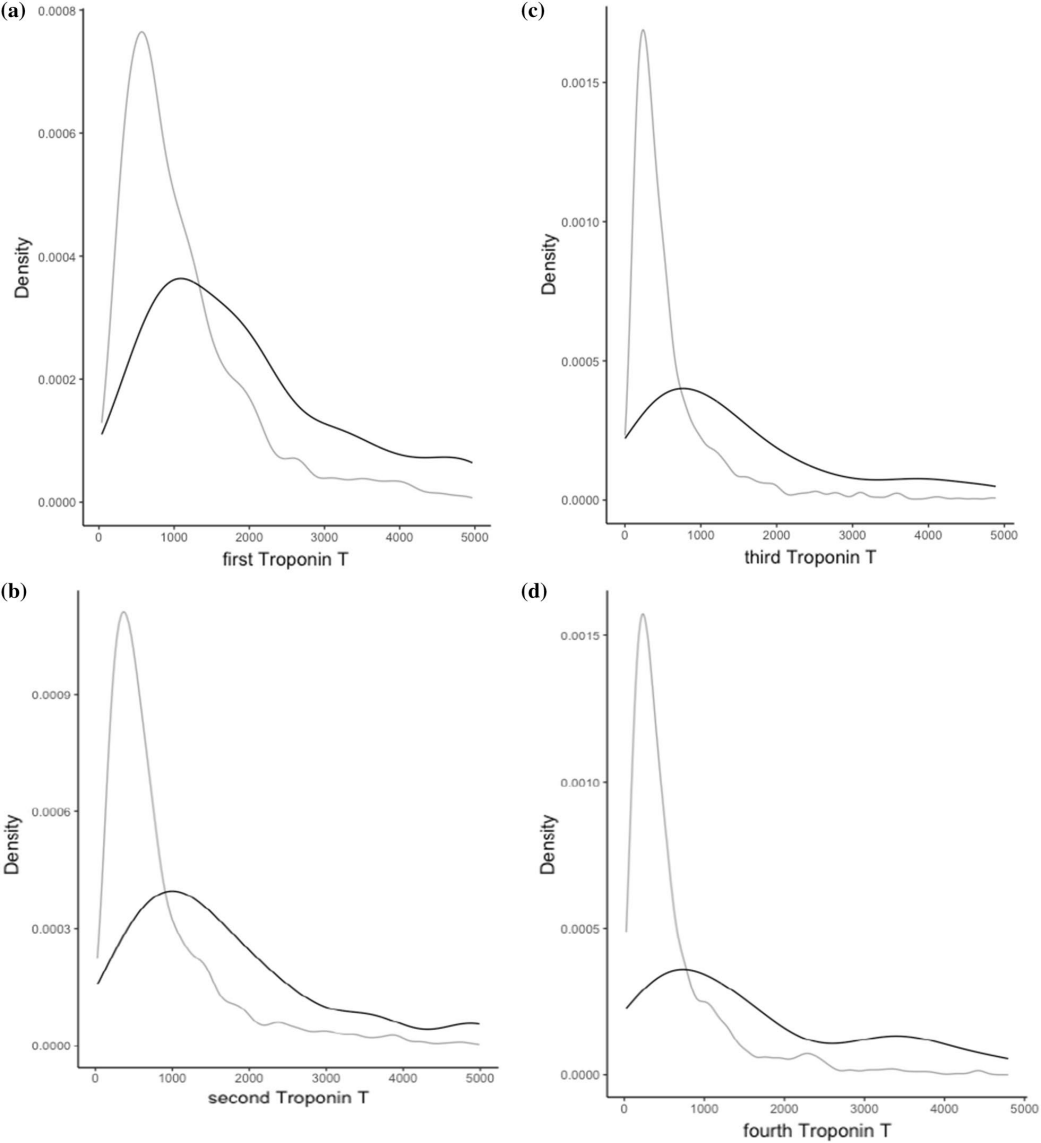
**a**–**d** Displays the density plot of the first to fourth hs troponins T, measured at d0 (**a**) immediately postoperative on admission at the ICU, d1 (**b**) first postoperative day at 6am (i.e.: 12–24 h postoperatively), d2 (**c**) second postoperative day at 6 am (i.e. 48–72 h postoperatively), and at d3 (**d**) third postoperative day at 6am (i.e. 96–120 h postoperatively). The black curve represents hs troponin T in deceased, the grey curve in surviving patients

This retrospective analysis included 2179 patients who underwent elective and urgent cardiac surgery using CPB in the period from 2013 to 2021. In total 7576 troponins were measured at the predefined timepoints. A total of 100 (4.59%) patients died during hospital admission. Table 1 displays the baseline characteristics of the study population as well as hsTnT serum concentrations in survived and deceased patients at various time points. Total median [IQR] postoperative hsTnT serum concentrations were: first hsTnT d0 928.00 ng/L [544.00, 1622.50] (n = 2163), second hsTnT d1 606.00 ng/L [351.25, 1158.50] (n = 2018), third hsTnT d2 398.00 ng/L [235.00, 753.00] (n = 1969) and fourth hsTnT d3 405.00 ng/L [225.25, 813.50] (n = 1426), respectively. Higher hsTnT serum concentrations, measured at any postoperative timepoint, were associated with increased hospital mortality. (Model 1) (first hsTnT d0 (n = 2163): *OR 1.31; 95% CI (1.23–1.39); p* < *0.001;* second hsTnT d1 (n = 2018)*: OR 1.36; 95% CI (1.26–1.46); p* < *0.001;* third hsTnT d2 (n = 1969): *OR 1.48; 95% CI (1.34–1.64); p* < *0.001;* fourth hsTnT d3 (n = 1426): *OR 1.56; 95% CI (1.39–1.76); p* < *0.001*). (Fig. 1A–D display density plots of hsTnT separated by mortality.) This finding persisted after multivariable adjusting for SAPS3, mean lactate at postoperative d1 and maximum dosage of Norepinephrine at postoperative d1. (Model 2) (first hsTnT d0 (n = 2163): a*OR 1.15; 95% CI (1.07–1.23); p* < *0.001;* second hsTnT d1 (n = 2018)*: aOR 1.21; 95% CI (1.12–1.31); p* < *0.001;* third hsTnT d2 (n = 1969): a*OR 1.27; 95% CI (1.15–1.40); p* < *0.001;* fourth hsTnT d3 (n = 1426): a*OR 1.34; 95% CI (1.18–1.53); p* < *0.001*). (Table 2) The type of the procedure (coronary artery bypass graft (CABG), aortic valve replacement (AVR) and other valve procedure) as well as the complexity of the procedure (defined as cross-clamp time) did not have any significant influence on the model performance and were therefore not included in Model 2 and the subsequent analysis.

**Table 1:** displays the baseline characteristics of the population and hsTnT serum concentrations in survived and deceased patients

	Deceased	Survived	*p*
Demographics			
n	100	2079	
Height at admission (cm) (mean (SD))	169.00 (9.48)	170.44 (11.24)	*0.206*
Weight at admission (kg) (mean (SD))	78.05 (17.93)	80.95 (19.46)	*0.144*
Age at admission (mean (SD))	71.45 (10.08)	66.95 (10.95)	< *0.001*
Sex = male (%)	38 (38.0)	550 (26.5)	*0.015*
Procedure			
Intervention CABG (n (%))	49 (49.0)	1161 (55.8)	*0.214*
Intervention AVR (n (%))	40 (40.0)	726 (34.9)	*0.351*
Intervention Valve (non AVR) (n (%))	54 (54.0)	976 (46.9)	*0.002*
Preexisting conditions			
Lung disease^a^ (n (%))	14 (14.0)	192 (9.2)	*0.157*
Diabetes (n (%))	29 (29.0)	421 (20.3)	*0.047*
Art. hypertension (n (%))	66 (66.0)	1359 (65.4)	*0.982*
Renal dysfunction (n (%))	37 (37.0)	323 (15.5)	< *0.001*
Premedication			
Betablocker (n (%))	52 (52.0)	1078 (51.9)	*1.000*
ACE inhibitor (n (%))	28 (28.0)	721 (34.7)	*0.205*
Statin (n (%))	52 (52.0)	1217 (58.5)	*0.234*
*Outcome*			
First hs Troponin T d0 (ng/L) (median [IQR])	2,103.00 [1147.00, 5,191.00]	898.00 [538.00, 1,554.00]	< *0.001*
Second hs Troponin T d1 (ng/L) (median [IQR])	2,012.00 [1049.75, 5,910.00]	590.00 [344.75, 1,092.00]	< *0.001*
Third hs Troponin T d2 (ng/L) (median [IQR])	1,818.00 [734.00, 5,674.00]	386.00 [233.00, 717.25]	< *0.001*
Fourth hs Troponin T d3(ng/L) (median [IQR])	1,501.00 [650.50, 3,731.00]	389.00 [219.50, 747.00]	< *0.001*
AKI KDIGO within first 48 h (n (%))			< *0.001*
KDIGO 0	16 (16.0)	40 (1.9)	
KDIGO 1	2 (2.0)	296 (14.2)	
KDIGO 2	30 (30.0)	1670 (80.3)	
KDIGO 3	52 (52.0)	73 (3.5)	
Norepinephrine max. dose (µg/kg/min) (mean (SD))	0.38 (0.28)	0.15 (0.13)	< *0.001*
Days of stay at ICU (mean (SD))	12.86 (15.38)	5.59 (7.18)	< *0.001*
Lactate avg. at d0 (mmol/L) (mean (SD))	3.67 (2.58)	1.69 (0.73)	< *0.001*
Lactate avg. at d1 (mmol/L) (mean (SD))	3.24 (3.25)	1.41 (0.50)	< *0.001*
SAPS3 (mean (SD))	59.12 (15.70)	43.07 (10.30)	< *0.001*
Cross clamp time (min) (mean (SD))	103.73 (67.66)	72.64 (39.82)	< *0.001*
CPB time (min) (mean (SD))	211.02 (111.68)	119.81 (59.56)	< *0.001*

Values are presented in mean (SD) or median [IQR] as appropriate due to distribution. Dichotomous variables are counted in n (%). Please note that more than one procedure can have been performed in one patient

*AVR* aortic valve replacement, *CABG* coronary artery bypass grafting, *AKI* acute kidney Injury, *KDIGO* Kidney Disease: Improving Global Outcomes classes 1–4, *CPB* Cardio Pulmonary Bypass

^a^Includes COPD, bronchial asthma, autoimmune diseases with pulmonary involvement, and pulmonary involvement in oncological diseases

**Table 2:** Displays the detailed results of model 2 for the first to fourth hs Troponin measurements

Variable	Crude OR (95% CI)	Adj. OR (95% CI)	P (Wald's test)	P (LR-test)	AIC value
First hs Troponin T d0 (ng/L)	1.3 (1.22, 1.38)	1.15 (1.07, 1.23)	< 0.001	< 0.001	
SAPS3	1.09 (1.07, 1.11)	1.05 (1.03, 1.07)	< 0.001	< 0.001	
Lactate avg. (mmol/L)	3.8 (2.98, 4.83)	2.05 (1.55, 2.72)	< 0.001	< 0.001	
Norepinephrine max. dose (µg/kg/min)	1.0041 (1.0033, 1.0048)	1.0016 (1.0007, 1.0025)	< 0.001	< 0.001	486.32
Second hs Troponin T d1 (ng/L)	1.35 (1.25, 1.45)	1.21 (1.12, 1.31)	< 0.001	< 0.001	
SAPS3	1.09 (1.07, 1.11)	1.05 (1.03, 1.07)	< 0.001	< 0.001	
Lactate avg. (mmol/L)	3.84 (2.94, 5.02)	1.96 (1.40, 2.73)	< 0.001	< 0.001	
Norepinephrine max. dose (µg/kg/min)	1.0039 (1.0031, 1.0047)	1.0015 (1.0005, 1.0025)	0.003	0.004	410.84
Third hs Troponin T d2 (ng/L)	1.48 (1.34, 1.64)	1.27 (1.15, 1.40)	< 0.001	< 0.001	
SAPS3	1.09 (1.07, 1.11)	1.05 (1.02, 1.07)	< 0.001	< 0.001	
Lactate avg. (mmol/L)	3.70 (2.80, 4.88)	1.81 (1.26, 2.59)	0.001	< 0.001	
Norepinephrine max. dose (µg/kg/min)	1.0037 (1.0029, 1.0046)	1.0015 (1.0004, 1.0026)	0.006	0.006	386.88
Forth hs Troponin T d3 (ng/L)	1.56 (1.39, 1.76)	1.34 (1.18, 1.53)	< 0.001	< 0.001	
SAPS3	1.08 (1.06, 1.1)	1.05 (1.03, 1.07)	< 0.001	< 0.001	
Lactate avg. (mmol/L)	3.16 (2.43, 4.1)	1.72 (1.23, 2.41)	0.002	0.001	
Norepinephrine max. dose (µg/kg/min)	1.0031 (1.0023, 1.004)	1.0011 (1.0001, 1.0022)	0.03	0.029	406.79

**Fig. 2:**
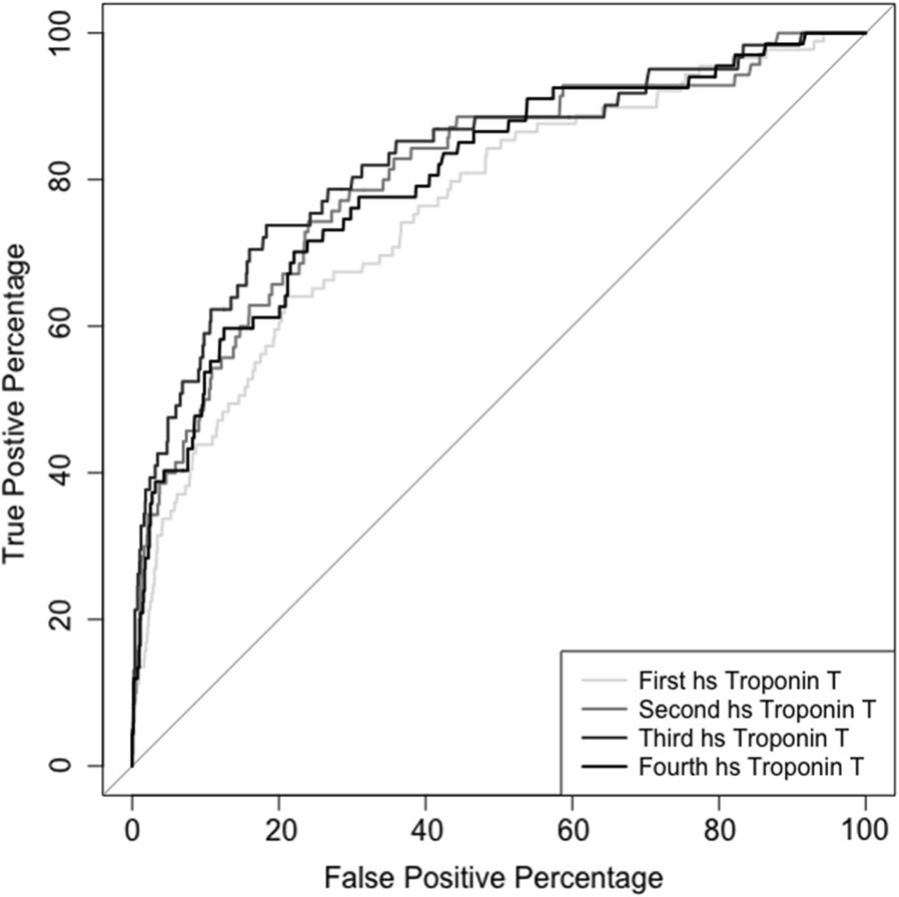
Displays the ROC analysis of the first to fourth hs troponin T for the uncorrected logistic regression model (Model 1), measured at d0 (first hs troponin T)—immediately postoperative on admission at the ICU, d1 (second hs troponin T)—first postoperative day at 6am (i.e.: 12–24 h postoperatively), d2 (third hs troponin T)—second postoperative day at 6 am (i.e. 48–72 h postoperatively), and at d3 (fourth hs troponin T)—third postoperative day at 6am (i.e. 96–120 h postoperatively). The AUC are as follows: First hs troponin T*: AUC 76.34%; 95% CI (0.71–0.82),* second hs troponin T: *AUC 80.77%; 95% CI (0.75–0.86)*, third hs troponin T: *AUC 82.74%; 95% CI (0.77–0.89)*, and fourth hs troponin T: *AUC 80.11%; 95% CI (0.74–0.86)*

**Fig. 3:**
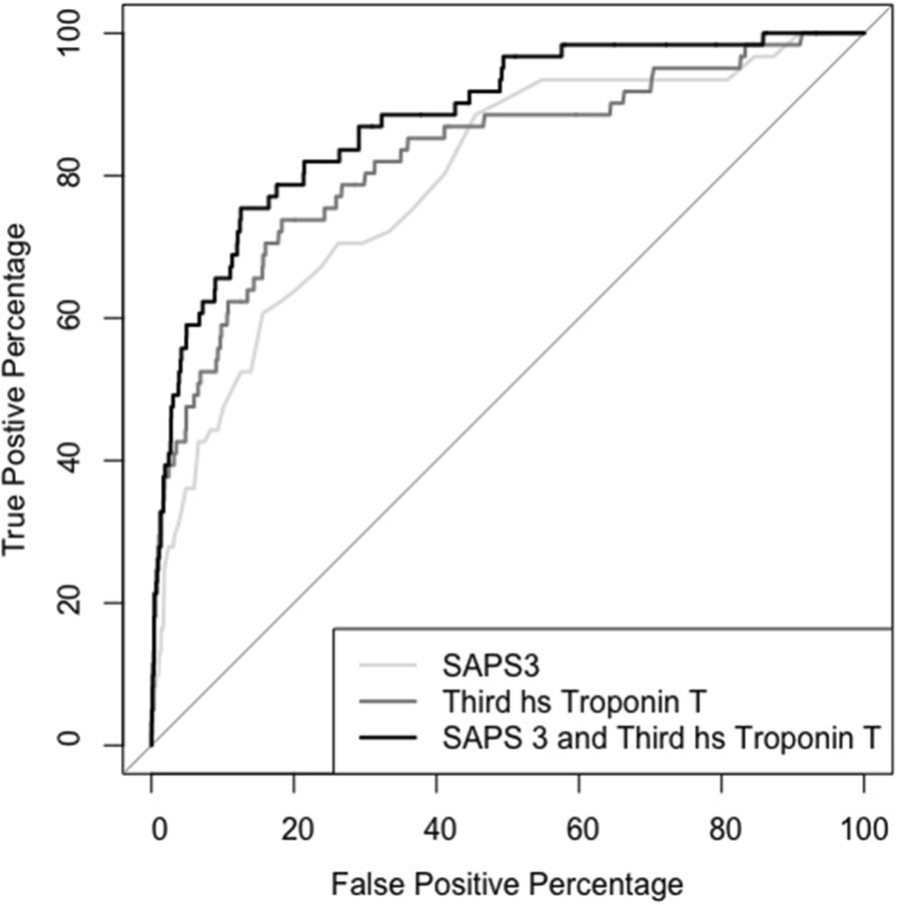
Displays the ROC analysis of the Simplified Acute Physiology Score 3 (SAPS3), third hs troponins T [measured at the second postoperative day at 6 am (i.e. 48–72 h postoperatively)] and the combined ROC analysis of SAPS3 and third hs troponins T. The AUC are as follows: SAPS3: *AUC 79.36%; 95% CI (0.73–0.85),* third hs troponin T: *AUC 82.74%; 95% CI (0.77–0.89)* and SAPS3 and third hs troponin T (combined): *AUC 87.96%; 95% CI (0.83–0.92).* The third hs troponin T was not superior to predict in-hospital mortality compared to SAPS3 *(IDI: 0.07; 95% CI [− 0.00 to 0.15]; p* = *0.057)*. However, adding the third hs troponin T to SAPS3 improved the prognostic accuracy compared to SAPS3 alone significantly *(IDI: 0.11; 95%CI [0.05–0.18]; p* < *0.001)*

ROC-analysis was performed to predict in-hospital mortality by hsTnT at all four predefined timepoints. Figure 2 displays the ROC-curves and the AUC for the base model (Model 1). The third hsTnT was shown to predict mortality with the highest precision. *(AUC 82.74%; 95% CI (0.77–0.89).* There was even a trend that the prediction of in-hospital mortality was better than the prediction by the SAPS3 score, however this was not statistically significant. *(AUC 79.36%; 95% CI (0.73–0.85); p* = *0.056).* Using the SAPS3 score as reference, the addition of hsTnT increased the AUC to predict in-hospital death *(AUC 87.96%; 95% CI (0.83–0.92); p* < *0.001)* (Fig. 3).

The optimal cut-off for the third hsTnT d2 was calculated to be 1264 ng/L *(Sensitivity 0.62; Specificity 0.88)* by means of the Youden-Index. Dividing the group by this threshold (hsTnT > 1264 ng/L) resulted in a mortality rate of 14.5% vs. 1.3% *(p* < *0.001)* and therefore in a statistically highly significant increase of the in-hospital mortality *(OR 12.42; 95% CI (7.27–21.23); p* < *0.001).* Table 3 displays main outcome parameters above and below the calculated threshold. The ability to discriminate mortality by the hsTnT threshold persisted after multivariable adjustment for SAPS3 *(aOR 6.9; 95% CI (3.55–13.4); p* < *0.001).*

**Table 3:** Displays relevant demographics and outcome parameters dived into two groups above the calculated threshold (HsTnT > 1264 ng/L) by the means of Youden Index

	hs Troponin T < 1264 ng/L at d2 after surgery	hs Troponin T > 1264 ng/L at d2 after surgery	
n	1707	262	p
Height on admission (cm) (mean (SD))	170.38 (11.20)	170.02 (12.67)	0.630
Weight on admission (kg) (mean (SD))	81.05 (19.40)	81.15 (20.61)	0.941
Age on admission (mean (SD))	67.28 (10.71)	67.77 (10.95)	0.493
Sex = male (n (%))	438 (25.7)	76 (29.0)	0.283
Procedure			
Intervention CABG (n (%))	976 (57.2)	138 (52.7)	0.193
Intervention AVR (n (%))	632 (37.0)	71 (27.1)	0.002
Intervention Valve (non AVR) (n (%))	808 (47.3)	148 (56.5)	< 0.001
Preexisting conditions			
Lung Disease (n (%))	170 (10.0)	21 (8.0)	0.380
Diabetes (n (%))	363 (21.3)	52 (19.8)	0.658
Art. hypertension (n (%))	1145 (67.1)	168 (64.1)	0.382
Renal dysfunction (n (%))	254 (14.9)	70 (26.7)	< 0.001
Premedication			
Betablocker (n (%))	912 (53.4)	140 (53.4)	1.000
ACE inhibitor (n (%))	614 (36.0)	81 (30.9)	0.127
Statin (n (%))	1042 (61.0)	137 (52.3)	0.009
Outcome			
Deceased (n (%))	23 (1.3)	38 (14.5)	< 0.001
First hs Troponin T d0 (ng/L) (median [IQR])	800.00 [505.50, 1,291.00]	3387.00 [2064.75, 5,190.25]	< 0.001
Second hs Troponin T d1 (ng/L) (median [IQR])	529.00 [326.00, 841.00]	2989.00 [2056.25, 4,729.75]	< 0.001
Third hs Troponin T d2 (ng/L) (median [IQR])	346.00 [218.00, 554.00]	2006.50 [1539.75, 3,538.25]	< 0.001
Fourth hs Troponin T d3 (ng/L) (median [IQR])	323.00 [199.00, 522.25]	1779.50 [1284.00, 2,826.00]	< 0.001
AKI KDIGO within first 48 h (n (%))			< 0.001
KDIGO 0	24 (1.4)	1 (0.4)	
KDIGO 1	251 (14.7)	24 (9.2)	
KDIGO 2	1385 (81.1)	183 (69.8)	
KDIGO 3	47 (2.8)	54 (20.6)	
Norepinephrine max. dose (µg/kg/min) (mean (SD))	0.14 (0.12)	0.24 (0.21)	< *0.001*
Days of Stay at ICU (mean (SD))	4.96 (6.03)	10.68 (10.97)	< 0.001
Lactate avg. at d0 (mmol/L) (mean (SD))	1.61 (0.58)	2.26 (1.43)	< 0.001
Lactate avg. at d1 (mmol/L) (mean (SD))	1.38 (0.45)	1.95 (1.26)	< 0.001
SAPS3 (mean (SD))	42.39 (9.84)	48.24 (13.21)	< 0.001
Cross Clamp Time (min) (mean (SD))	69.12 (35.53)	100.67 (54.92)	< 0.001
CPB Time (min) (mean (SD))	113.86 (52.94)	171.07 (83.36)	< 0.001

Values are presented in mean (SD) or median [IQR] as appropriate due to distribution. Dichotomous variables are counted in n (%)

*AVR* aortic valve replacement, *CABP* coronary artery bypass grafting, *AKI* acute kidney Injury, *KDIGO* Kidney Disease: Improving Global Outcomes classes 1–4, *CPB* Cardio Pulmonary Bypass)

## Discussion

This study shows that elevated hsTnT levels in the postoperative period after cardiac surgery are (1) associated with an increased risk of death, (2) suitable to predict mortality after cardiac surgery similar to the SAPS3 score, and (3) have an ideal threshold of 1,264 ng/L at d2 after surgery to identify increased risk of death. The first finding was somewhat expected as many previous studies [10, 11, 14], including a more than 10 year old metanalysis, were able to show a correlation between any myocardial enzyme (creatine kinase (CK-MB), troponin, or both) elevation and survival following CABG [7]. However, the second two findings include a profound novelty: Previously, early postoperative troponin serum concentrations (a few hours after surgery) were primarily analyzed to predict mortality in cardiac surgery [14, 19]. To our knowledge only one large study identified the postoperative levels of hsTnI on d2 and d3 as ideal timepoints to predict mortality [10]. In this very recent study, Devereaux et al. used a hsTnI assay (ARCHITECT STAT^®^ (Abbott Laboratories); upper reference limit 26 ng/L), which differs from our assay (ROCHE Elecsys^®^ Troponin T high sensitive (TnT-hs); upper reference limit 14 ng/L). In clinical practice, there seems to be little difference in the use between hsTnI and hsTnT, as the choice hs-Tn assay primarily depends on commercial availability. However, even though there are similarities, hsTnI and hsTnT are specific to each high-sensitivity cardiac troponin assay and cannot be generalized to other cardiac troponin assays. Devereaux et al. were able to identify different thresholds in hsTnI for different types of surgery ranging from 5670 ng/L to 1522 ng/L in CABG and AVR and up to 12,981 ng/L to 2503 ng/L in other procedures on the first and second/third day, respectively. In contrast, we did not investigate the type of surgery further. In our multivariate modeling (Model 2) the type of procedure (CABP, AVR, and other valve procedure) did not affect the performance of the model and the type of procedure therefore was excluded in the backward variable selection. Interestingly, our analysis showed that the optimal time to determine hsTnT blurred after multivariate correction. Similar to Devereaux et al., the identified cutoff value in this study (exceeding 90 times the upper reference limit of the assay) was far higher than previously accepted thresholds, established by various consensus statements (ranging somewhere from > 10 to a maximum of ≥ 70 times the upper reference limit of an assay) [12, 13]. Addressing the lack of a missing threshold for postoperative TnT, Gahl et al. analyzed the association between an early postoperative hsTnT level of > 800 ng/L (6–12 h postoperatively) and major adverse cardiac or cerebrovascular events (MACCE) [14]. The authors found that the risk of MACCE significantly increases above the respective hsTnT threshold. A lower threshold value in earlier postoperative hsTnT measurements was consistent with our data. For illustration purposes we calculated the optimal threshold in our second hsTnT, which was 1127 ng/L *(Sensitivity 0.74, Specificity 0.76)* for in-hospital mortality (not MACCE). Arguably earlier measured postoperative hsTnT serum concentrations do result in a lower threshold to predict any form of negative outcome. We believe this is mainly due to the influence of intraoperative occurrences, such as intraoperative direct trauma to the myocardium or the procedure itself. Ultimately, it remains unclear to what extend inconsistencies in the thresholds for clinically important perioperative myocardial injury are attributed to the peculiarity of the cardiac biomarker, the preceding procedure or the timing when the samples were obtained.

Several limitations of our study need to be considered: Given the observational nature of our data, the lack of randomization does not allow for any causal conclusions, but rather careful consideration and interpretation of associations. Despite significant efforts to adjust for baseline differences, the presence of residual confounding factors cannot be ruled out given the observational nature. Hence, the interpretation of any cutoff or threshold value has to be done with caution. When designing our models, we were careful to avoid multicollinearity and used caution in the variable selection. After including the SAPS3 score, we deliberately did not include any additional parameters of renal function (such as AKI class 3 as described by the definition of the acute kidney injury work group (Kidney Disease: Improving Global Outcomes—KDIGO), serum creatinine, or bun). Due to lack of detailed information in our data we could not correct for type and sufficiency of cardioplegia or potential procedures per se producing elevated troponin (e.g.: Maze procedure). However, we attempted to incorporate age and sex into the models, but found that those did not improve the quality of the prediction. Our estimates of hsTnT thresholds are based on an increased risk of in-hospital death. Long-term outcomes (such as one-year mortality) may also be influenced by the extent of myocardial injury, and it is possible that the threshold of myocardial injury for such outcomes may be lower than those we have identified. Our threshold estimates were done by means of a Youden analysis for the whole ROC Analysis. We did not weigh for high sensitivity or high specificity. Therefore, the threshold is subject to considerable uncertainty, as indicated by the relatively low sensitivity of only 0.62. Ultimately, due to the sample size and concerns for precision we did not attempt to split our dataset to generate a separate validation dataset. Our findings provide the basis for further research and validation on the accurate prediction of in-hospital mortality in patients undergoing cardiac surgery using biomarkers.

## Conclusion

This study examined whether postoperative hsTnT measurement is suitable for the prediction of in-hospital mortality in patients undergoing cardiac surgery. We found that hsTnT on postoperative day two and three had the best association to in-hospital mortality and was therefore best suited for the prediction of mortality. The precision of the prediction of in-hospital mortality by hsTnT was comparable to the SAPS3 score. Additionally, we found that the lowest threshold values of hsTnT associated with an increased incidence of in-hospital death from any cause were markedly higher than thresholds currently recommended by consensus statements for the detection of perioperative myocardial infarction and clinically important perioperative myocardial injury.

### Supplementary Information

**Additional file1: Appendix 1. **The RECORD statement—checklist of items, extended from the STROBE statement, that should be reported in observational studies using routinely collected health data.

## Data Availability

The SICdb dataset is publicly available on PhysioNet (Ref. #15) However, ‘contributed approval’ access is currently in place.
