# Supplementary material for: Timing and threshold of high sensitive troponin T measurement for the prediction of mortality after cardiac surgery: a retrospective cohort analysis
Source: Intensive Care Med Exp. 2023 Sep 1;11:58. doi: 10.1186/s40635-023-00545-z (PMC10473995; doi:10.1186/s40635-023-00545-z)
Supplement: Supplementary file 1 — Additional file1: Appendix 1. The RECORD statement—checklist of items, extended from the STROBE statement, that should be reported in observational studies using routinely collected health data. [file 40635_2023_545_MOESM1_ESM.docx]

**The RECORD statement – checklist of items, extended from the STROBE statement, that should be reported in observational studies using routinely collected health data.**

|  | **Item No.** | **STROBE items** | **Location in manuscript where items are reported** | **RECORD items** | **Location in manuscript where items are reported** |
| --- | --- | --- | --- | --- | --- |
| **Title and abstract** | | | | | |
|  | 1 | (a)/(b) We performed a retrospective single-institutional cohort analysis of 2,179 patients undergoing elective and urgent cardiac surgery with cardiopulmonary bypass from 2013 to 2021. Logistic regression analysis was used to investigate an association of hsTnT at various time points and in-hospital mortality. Results were compared to the SAPS3 score. | 1 (a) and (b) can be found in the abstract/methods. | RECORD 1.1: No pre-existing dataset was used. Data source is described in the abstract.  RECORD 1.2: As the geographic location need a somewhat longer explanation this part was moved from the abstract to the methods section  RECORD 1.3: only one data source/database was used. | 1.1 can be found in the abstract/methods  1.2 and 1.3 need a longer explanation, therefore both can be found in the study design section in methods. |
| **Introduction** | | | | | |
| Background rationale | 2 | This study sought to investigate the applicability of sequential postoperative hsTnT measurements to predict ICU mortality after cardiac surgery using cardiopulmonary bypass (CPB). We hypothesized, that later hsTnT measurements at postoperative day 2 (d2) or 3 (d3) are superior to predict mortality compared to early measurements at the day of surgery (d0) or first day after surgery (d1). Additionally, we determined a cut point to detect increased mortality by hsTnT. | To be found in the introduction. |  |  |
| Objectives | 3 | We hypothesized, that later hsTnT measurements at postoperative day 2 (d2) or 3 (d3) are superior to predict mortality compared to early measurements at the day of surgery (d0) or first day after surgery (d1). | To be found in the introduction. |  |  |
| **Methods** | | | | | |
| Study Design | 4 | This retrospective cohort analysis included patients who underwent elective and urgent cardiac surgery utilizing CPB from 2013 to 2021. The exposure of interest was mortality during hospital stay. | To be found in the in the study design section in methods. |  |  |
| Setting | 5 | General Hospital Salzburg, Paracelsus Medical University; 2013 to 2021; Follow up was ‘stay at the hospital’; Data was extracted from the institutional Salzburg Intensive Care database (SICdb) which was recently developed. SICdb was implemented using data from the hospital ICU data management system (PDMS) iMDsoft MetaVision ICU (iMDsoft, Needham, MA) and the electronic health record (EHR) ORBIS (DH Healthcare GmbH, Bonn, Germany). The database contains admission, discharge, procedural and ICD10 data. Additionally, all medications (including dosage), procedures such as renal replacement therapy (RRT), intubation etc. are reported in SICdb. | To be found in the in the study design section in methods. |  |  |
| Participants | 6 | *(a) Cohort study* – All patients undergoing cardiac surgery in the period described above were included in the analysis. Every patient undergoing Cardiac surgery (elective and urged) at the institution were recorded using an PDMS electronic health record. The follow up was during the whole stay at the hospital.  *(b) Case-control study* – N.a. | To be found in the in the study design section in methods. | RECORD 6.1: There was no defined process for patient selection. All patients undergoing cardiac surgery were included.  RECORD 6.2: N.a.  RECORD 6.3: N.a. | 6.1 to be found in the in the study design section in methods. |
| Variables | 7 | The primary outcome of the study was to define the ideal a postoperative time point to measure hsTnT for mortality prediction. Plasmatic hsTnT concentrations were measured using the ROCHE Elecsys® Troponin T high sensitive (TnT-hs) assay. Predefined time points for the measurements were: 1st hsTnT at d0 – immediately postoperative on admission at the ICU, 2nd hsTnT at d1 – first postoperative day at 6am (i.e.: 12-24h postoperatively), 3rd hsTnT at d2 – second postoperative day at 6 am (i.e. 48-72h postoperatively), 4th hsTnT at d3 – third postoperative day at 6am (i.e. 96-120h postoperatively). Secondary outcome was the ideal cutoff value for postoperative hsTnT to predict mortality and a comparison to the SAPS3 score | To be found in the in the endpoints and statistical analysis section in methods. | RECORD 7.1: All models and logistic regressions were done with R Project for Statistical Computing (RCore Team, 2022). Results were visualized using R Studio (RStudio Team, 2022, Boston, MA). Model 2 was defined as: Model 2 <- glm(Deceased ~ TroponinTThird + SAPS3 + LactAvgD1 + Norep_yKgMin_MaxD1, data = DDAVP, family = "binomial") | Detailed description of the models can be found in the statistical analysis section in methods. The full Results of model 2 are displayed in Table 4 |
| Data sources/ measurement | 8 | All used variables were electronically recorded using the hospital ICU data management system (PDMS) iMDsoft MetaVision ICU (iMDsoft, Needham, MA) and the electronic health record (EHR) ORBIS (DH Healthcare GmbH, Bonn, Germany). No other non-electronically recorded data was used. | To be found in the in the study design section in methods. |  |  |
| Bias | 9 | We do not see any relevant source of bias; However, several limitations were reported in the discussion section | To be found in the in the discussion. |  |  |
| Study size | 10 | This retrospective analysis included 2,179 patients and 7,576 hsTnT measurements (first hsTnT n=2,163; second hsTnT n=2,018; third hsTnT n=1,969; fourth hsTnT, n=1,426). | To be found in the in the study design section in methods. |  |  |
| Quantitative variables | 11 | N.a. |  |  |  |
| Statistical methods | 12 | (a) We expressed continuous data points as median ± interquartile range or mean ± SD. Categorical data were stated in numbers (percentage). Univariate distribution differences between groups were calculated using the Chi-square test and one-way ANOVA, respectively.  Logistic regression analysis was used for the primary binary outcome ‘in-hospital mortality’. (Model 1) We did report the SAPS 3 score and other potential influencing patient characteristics (Table 1) For the multivariate regression model, cofounders with a p-value <0.10 in the univariate analysis were included, then a backward variable elimination was performed. Elimination criterion was a p-value >0.10. ROC analysis and AUC calculation was done to predict in-hospital mortality by hsTnT (Model2). ROC-analysis was performed to predict in-hospital mortality by hsTnT at all four predefined timepoints. We reported adjusted odds ratios (aOR) with respective 95% confidence intervals (95%CI) for all models. We additionally assessed the corresponding integrated discrimination improvement (IDI) using the SAPS3 score as a reference. The IDI reflects of prognostic performance of hsTnT serum concentrations compared to the reference model and the gain in prognostic performance when adding the hsTnT serum concentrations to the reference model.  All statistical tests were two-sided, a p-value of <0.05 was considered statistically significant.  (b) N.a.  (c) Patients with missing hsTnT were excluded from the analysis. (n=1,140)  (d) *Cohort study* – There was no loss in follow up.  (e) N.a. | To be found in the in the statistical analysis section in methods. |  |  |
| Data access and cleaning methods |  |  |  | RECORD 12.1: All patients had surgery at the corresponding authors institution. Therefore, full access to all patients records and source database.  RECORD 12.2: N.a. | To be found in the in the study design section in methods. |
| Linkage |  |  |  | RECORD 12.3: N.a. |  |
| **Results** | | | | | |
| Participants | 13 | (a) 2,179 patients were analysed in total.  (b) N.a.  (c) N.a. | To be found in the in the study design and statistical analysis section in methods. | RECORD 13.1: This retrospective cohort analysis included patients who underwent elective and urgent cardiac surgery utilizing CPB at the General Hospital Salzburg, Paracelsus Medical University from 2013 to 2021. 2,179 patients were analysed. However due to missing data on hsTnT a total of 1,140 measurements are missing and only 7,576 hsTnT were included in the analysis. | To be found in the in the study design and statistical analysis section in methods. |
| Descriptive data | 14 | (a) All participants were scheduled for elective or urgent cardiac surgery.  (b) The descriptive analysis of the study population can be found in table 1 and 2, respectively.  (c) *Cohort study* – N.a. | To be found in the in the study design and statistical analysis section in methods, and in Table 2 and 2. |  |  |
| Outcome data | 15 | *Cohort study* - The descriptive analysis of the study population can be found in table 1 and 2, respectively.  *Case-control - N.a.*  *Cross-sectional study* – N.a. | To be found in the in the study design and statistical analysis section in methods, and in table 2 and 2. |  |  |
| Main results | 16 | (a) Higher hsTnT, measured at any postoperative timepoint, was associated with increased hospital mortality. (Model 1) (first hsTnT: *OR 1.31; 95% CI (1.23-1.39); p<0.001;* second hsTnT*: OR 1.36; 95% CI (1.26-1.46); p<0.001;* third hsTnT: *OR 1.48; 95% CI (1.34-1.64); p<0.001;* fourth hsTnT: *OR 1.56; 95% CI (1.39-1.76); p<0.001*). This finding persisted after multivariable adjusting for SAPS3, mean lactate at postoperative d1 and maximum dosage of Norepinephrine at postoperative d1. (Model 2) (first hsTnT: a*OR 1.15; 95% CI (1.07-1.23); p<0.001;* second hsTnT*: aOR 1.21; 95% CI (1.12-1.31); p<0.001;* third hsTnT: a*OR 1.27; 95% CI (1.15-1.40); p<0.001;* fourth hsTnT: a*OR 1.34; 95% CI (1.18-1.53); p<0.001*). The third hsTnT was shown to be able to predict mortality with the highest precision. *(AUC 0.82; 95% CI (0.77-0.89).* This prediction of in-hospital mortality was slightly, though not statistically significantly, better than the prediction by the SAPS3 score *(AUC 79.36%; 95% CI (0.73-0.85); p=0.056).* Using the SAPS3 as reference, the addition of hsTnT increased the AUC to predict in-hospital death *(AUC 87.96%; 95% CI (0.83-0.92); p<0.001).* The optimal cut-off for the third hsTnT was calculated to be 1,264ng/L *(Sensitivity 0.62; Specificity 0.88)* by means of the Youden-Index.  (b) Report category boundaries – N.a.  (c) N.a. | To be found in the abstract and results section. |  |  |
| Other analyses | 17 | Univariate distribution differences between groups were calculated using the Chi-square test and one-way ANOVA, respectively. | To be found in the in the study design and statistical analysis section in methods. results are displayed in table 2 |  |  |
| **Discussion** | | | | | |
| Key results | 18 | This study shows that elevated hsTnT levels in the early postoperative period after cardiac surgery are (1) associated with an increased risk of death, (2) suitable to predict mortality after cardiac surgery and (3) have an ideal cut point of 1.264ng/L at d2 after surgery to identify increased risk of death. | To be found in the abstract and in the results/discussion. |  |  |
| Limitations | 19 | An extensive discussion of all/the most common limitations of the study is provided in the discussion section: In conclusion limitations include but are not limited to: retrospective character of the study, single center study; | To be found in the abstract and in the discussion. | RECORD 19.1: Given the observational nature of our data, inherent limitations include the lack of randomization which does, as stated before, not allow for any casual conclusions, but rather careful consideration and interpretation of associations. Additional limitations of the study are described in the STROBE statement (#19) | To be found in the statistical methods in the methods section, the results and in the discussion. |
| Interpretation | 20 | Elevated hsTnT after cardiac surgery was associated with an increased risk of in-hospital mortality. HsTnT measured at postoperative day 2 and 3 had the highest power to predict in-hospital mortality. HsTnT levels currently recommended to define clinically important periprocedural myocardial injury are lower than thresholds identified in this study. | To be found in the abstract |  |  |
| Generalisability | 21 | We found that the lowest threshold values of hsTnT associated with increased rates of death from any cause was markedly higher than the threshold values recommended in consensus statements for the detection of perioperative myocardial infarction and clinically important perioperative myocardial injury. | To be found in the conclusion. |  |  |
| **Other Information** | | | | | |
| Funding | 22 | Solely, institutional funding, provided by the Department of Anaesthesiology, Perioperative Medicine and Intensive Care Medicine, Paracelsus Medical University of Salzburg, Austria. No (industry) sponsorship has been received for this investigator-initiated study. | To be found in the declaration of conflicts of interest. |  |  |
| Accessibility of protocol, raw data, and programming code |  |  |  | RECORD 22.1: The institutional Salzburg Intensive Care database (SICdb) is intended to be publicly published, using the repository for biological data PhysioNet. PhysioNet is a collaborative project and is supported by the National Institutes of Health (NIH) and other organizations. SICdb can be accessed on PhysioNet: Rodemund N, Kokoefer A, Wernly B, Cozowicz. C. “Salzburg Intensive Care database (SICdb), a freely accessible intensive care database” (version 1.0.5). *PhysioNet*. 2023. doi:https://doi.org/10.13026/67ww-6m34. | To be found partly in Methods. |

*Reference: Benchimol EI, Smeeth L, Guttmann A, Harron K, Moher D, Petersen I, Sørensen HT, von Elm E, Langan SM, the RECORD Working Committee. The REporting of studies Conducted using Observational Routinely-collected health Data (RECORD) Statement. *PLoS Medicine* 2015; in press.

*Checklist is protected under Creative Commons Attribution ([CC BY](http://creativecommons.org/licenses/by/4.0/)) license.
